# Can co-authorship networks be used to predict author research impact? A machine-learning based analysis within the field of degenerative cervical myelopathy research

**DOI:** 10.1371/journal.pone.0256997

**Published:** 2021-09-02

**Authors:** Noah Grodzinski, Ben Grodzinski, Benjamin M. Davies

**Affiliations:** 1 St John’s College, University of Cambridge, Cambridge, United Kingdom; 2 School of Clinical Medicine, University of Cambridge, Cambridge, United Kingdom; 3 Division of Neurosurgery, Department of Clinical Neurosciences, University of Cambridge, Cambridge, United Kingdom; University of Toronto, CANADA

## Abstract

**Introduction:**

Degenerative Cervical Myelopathy (DCM) is a common and disabling condition, with a relatively modest research capacity. In order to accelerate knowledge discovery, the AO Spine RECODE-DCM project has recently established the top priorities for DCM research. Uptake of these priorities within the research community will require their effective dissemination, which can be supported by identifying key opinion leaders (KOLs). In this paper, we aim to identify KOLs using artificial intelligence. We produce and explore a DCM co-authorship network, to characterise researchers’ impact within the research field.

**Methods:**

Through a bibliometric analysis of 1674 scientific papers in the DCM field, a co-authorship network was created. For each author, statistics about their connections to the co-authorship network (and so the nature of their collaboration) were generated. Using these connectedness statistics, a neural network was used to predict H-Index for each author (as a proxy for research impact). The neural network was retrospectively validated on an unseen author set.

**Results:**

DCM research is regionally clustered, with strong collaboration across some international borders (e.g., North America) but not others (e.g., Western Europe). In retrospective validation, the neural network achieves a correlation coefficient of 0.86 (p<0.0001) between the true and predicted H-Index of each author. Thus, author impact can be accurately predicted using only the nature of an author’s collaborations.

**Discussion:**

Analysis of the neural network shows that the nature of collaboration strongly impacts an author’s research visibility, and therefore suitability as a KOL. This also suggests greater collaboration within the DCM field could help to improve both individual research visibility and global synergy.

## Introduction

Degenerative Cervical Myelopathy (DCM) is an umbrella term that refers to symptomatic spinal cord compression secondary to degenerative changes of the cervical spine [1]. It is common, with an estimated prevalence of 2.3% [2]. Symptoms are lifelong, progressive, and disabling, with a significant impact on patient quality of life [3]. Despite this, awareness of the condition amongst healthcare professionals and the general public is low [4] and research that improves outcomes is urgently required [5].

Research prioritisation is a means of accelerating progress, by consolidating research investment on critical questions [4]. The AO Spine RECODE-DCM project has recently published the top priorities for DCM research [6]. To ensure that these priorities impact DCM research, it is crucial for research field to adopt these findings. This process is often referred to as knowledge translation (KT), or implementation science. One of common barriers to overcome is information sharing, and a recognised mechanism for doing so effectively revolves around identifying key individuals or organisations well positioned to distribute findings to a larger but relevant audience. These can be referred to as ‘agents of change’ (AoC) [7].

A crucial target audience for the AO Spine RECODE-DCM findings are those currently engaged with DCM research. However, DCM’s ‘research landscape’–its researchers, their connections, institutions, and countries–is poorly characterised. A recent evaluation was able to provide a shortlist of leading contributors, using existing bibliometrics and also through a network analysis [8], demonstrate regional clustering with limited connections. However, the significance of collaboration was not defined.

Collaboration is a corner stone of scientific progress, and likely to be an important part of answering these research priorities. To date the nature of collaboration within DCM has not been evaluated, nor its relationship on impact–for example, the existence of an optimum research group size, or the importance of international or inter-institutional collaboration. Characterising the DCM research landscape could hold value for knowledge translation, by uncovering the key AoC well positioned to facilitate KT in DCM.

Today, the most commonly used surrogate of an individual researcher’s impact is the H-Index [9], which takes into account both the numbers of papers an author has published, and the number of citations of those papers. However, H-Index may not be best suited to identifying AoCs, as it can misrepresent an author’s global visibility, due to self-citation or research activity in parallel fields [10]–an impact metric without these drawbacks is required.

In this study, we generate a DCM co-authorship network and use a machine learning (neural network) approach, to evaluate the nature and significance of collaboration with respect to scientific visibility.

## Methods

### Co-authorship network

#### Article selection and extraction of authorship

Within the field of DCM, there is inconsistent disease terminology, with an absence of index terms or codes [11, 12], which makes literature searching extremely inefficient. For this reason, and to support work by Myelopathy.org, an international charity for DCM, building on original systematic reviews [13, 14], a database of primary clinical trials exclusively related to DCM has been maintained [15]. This is based on a search of EMBASE and MEDLINE for ‘Cervical’ and ‘Myelopathy’, and hand searching for primary clinical studies exclusively related to DCM from 1995 (e.g., reviews or editorials are not included). In accordance with PRISMA guidelines, titles and abstracts were initially screened, and subsequently full-text papers were screened and extracted by four reviewers over multiple iterations. At the most recent update on 8 August 2020, it contained 1674 articles.

Using the Pybliometrics programme, this database was cross referenced with Scopus [16] to identify author details and their respective H-Index. Scopus collates information on authors using unique Electronic IDs (EIDs), rather than non-unique names [17].

Using this script, 1281 papers and 4317 authors were identified, based on a unique match using paper title. For each author, the number of papers published in DCM was calculated. The H-Index of author, the total number of citations of all the author’s papers, and the total number of author’s papers were pulled from the Scopus database, along with the current country and affiliation of the author. 16,888 unique co-authorship links were created from all the papers.

#### Author selection and network formation

In order to reduce noise and select authors more focused and active within DCM research, only authors with at least five papers in the field of DCM *or* with at least two papers in DCM, and where DCM forms at least 10% of their overall research output, were included. Additionally, authors without any co-authorship links were excluded.

These criteria were defined by reference to the original set of 4317 authors–many formed isolated clusters of researchers, whose research was almost entirely tangential to DCM, and who had published only one paper within the DCM field. These authors’ H-Index values would therefore have been a poor reflection of their research impact specifically in DCM and so they were excluded. Authors with above five papers were considered “prolific”, so included. So as not to discriminate against newer authors who have simply published fewer papers overall, authors who had less than 5 papers (but still 2 or more) were included, if above 10% of their overall number of papers were on the topic of DCM, and so a reasonable proportion of their research output.

With these criteria in place, 543 of the original 4317 authors were included in the final network, forming 2309 unique co-authorship links, using the iGraph library (version 1.2.6) [18] in R (version 4.0.3).

### Machine learning

In order to predict research impact of authors, a quantitative surrogate for research impact of each author was required, to provide a predictive variable for the machine-learning program. For this purpose, H-Index [9] of author was chosen. The H-Index of an author is the largest number of papers that author has published with at least that many citations.

H-Index was chosen as the most suitable surrogate impact metric for a number of reasons. First, its wide adoption in many academic fields, as a measure of both quality and quantity of an author’s research, maximises the generalisability of this method, and means that H-Index values are readily available for each author. Second, the range of values of H-Index is fairly small (rarely exceeding 100). This means that absolute differences in H-Index remain meaningful across the entire spectrum of values (unlike total citation count, where the difference between 10 and 15 citations is very different from 10,000 and 10,005). This is more suitable for the machine learning structure used (described below). Third, single papers with many citations are not over-emphasized. This means that inconsistent authors, or co-authors of a single influential paper, are not overemphasized in impact. No other quantitative metrics were found which incorporated both the quantity and quality of research and were also widely available for each author. Qualitative metrics would not be suitable for the machine learning approach employed in this paper.

#### Machine learning model

A simple feedforward multilayer perceptron was used as the structure of the neural network for machine learning. This is a general standard for pattern recognition. The cost function for each batch was the mean square error, where the error is the difference between the true and predicted result (author H-Index). The activation function of all neurons was the RALU function. These are standard choices for simple neural networks [19] for regression.

The neural network had 13 input neurons, corresponding to the 13 pieces of network data generated for each author. There were 2 hidden layers (for basic pattern recognition; more hidden layers would have likely resulted in “overfitting”, or the neural network “memorising” the data given [20]) consisting of 64 then 32 neurons, and then an output layer with a single neuron (to indicate H-Index prediction). The final neuron output was interpreted as the predicted H-Index.

**Fig 1:**
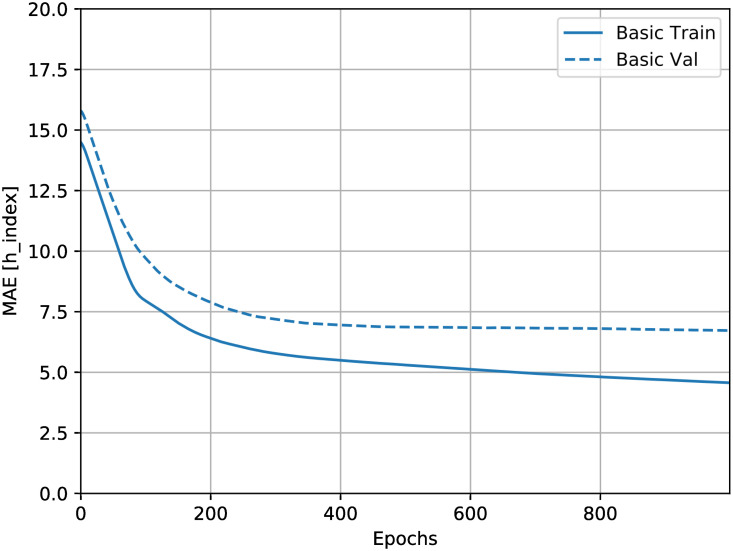
Mean average error over 1000 training epochs. Mean average error of the neural network, i.e. the average difference between the true and predicted author H-Index, for each epoch. After around 400 epochs, the network starts to overfit to the training data, as the training error rate continues to go down, while the validation error rate (shown as a dotted line) remains roughly constant.

The neural network code itself was generated from TensorFlow [21]. The batch size was 32, and the network was trained over 1000 epochs, found to be the most effective to prevent over-fitting but to still maximise training (Fig 1).

#### Statistical analysis

The neural network was trained on 80% of the authors, leaving 20% for prospective validation (randomly chosen from the dataset).

After training, the neural network generated predictions of H-Index values for the remaining 20% of authors (109). These values were then compared to the true H-Index values using Spearman’s rank correlation coefficient to test for positive correlation between true and predicted H-Index. Spearman’s rank correlation coefficient was used as H-Index was not normally distributed, and as the key prediction is relative author impact instead of absolute values. The accuracy of the network was also measured.

Finally, the sensitivity of the network to different input variables was measured. This gives an indication as to how an author should collaborate in order to maximise their H-Index.

## Results

### Co-authorship network

**Fig 2:**
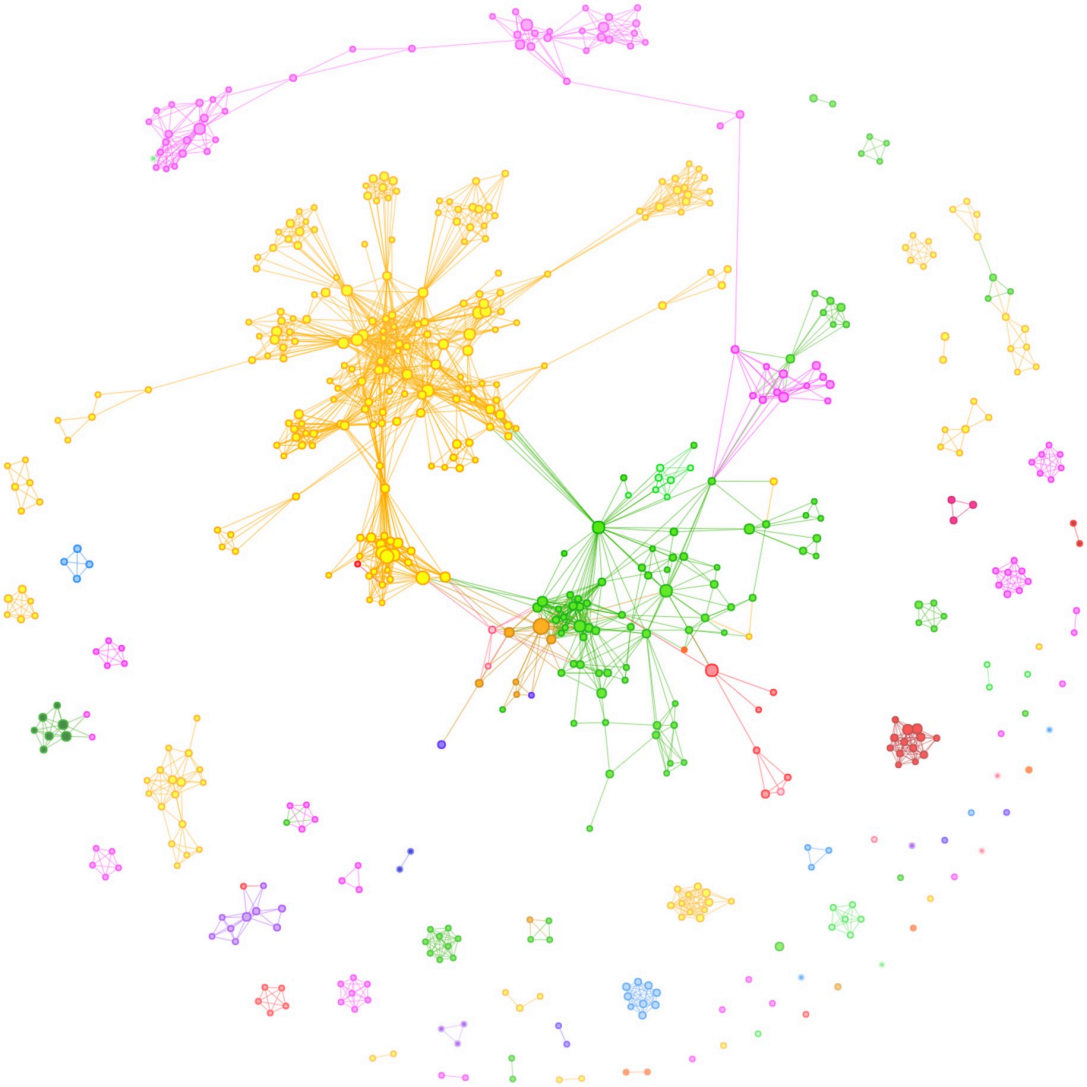
Visualisation of co-authorship network for DCM research. Colours show country. Author node size shows number of papers published. Authors shown as lone nodes do not have any collaborations with the other “prolific” selected authors.

The co-authorship network is displayed in Fig 2, and an interactive version is available within the supporting information (S1 Fig).

As identified previously [8], and based upon visual inspection, clusters are largely explained by national boundaries–cross-border connections (i.e., collaborations) are more limited. The main areas of international collaboration are within North America with links between American and Canadian centres (e.g. Riew K.–Fehlings M.), and between North America and Japan (e.g. Riew K.–Oshima M.; Fehlings M.–Nakashima H.). International collaboration across other continents (e.g. Europe) is much more limited.

95 of the included authors had at collaborations with at least 1 other country, representing only 17.5% of all included authors. By contrast, 95% of authors collaborated with at least 1 other research institution in their research, and 49% of authors collaborated with authors in a total of at least 5 research institutions.

Using the co-authorship network, network involvement statistics were generated for each author (Table 1).

**Table 1:** Top 10 authors, by various network measures.

*Top 10 authors by H-Index*		*Top 10 authors by 1st Order Connections*	
**H-Index**	**Name of author**	**Name of author**	**Connections**
94	Fehlings M.	Seichi A.	45
84	Vaccaro A.	Takeshita K.	42
76	Yoshikawa H.	Taguchi T.	39
76	Kawaguchi H.	Kato T.	36
73	Toyama Y.	Toyama Y.	36
70	Shaffrey C.	Riew K.	36
67	McGirt M.	Yamazaki M.	33
67	Albert T.	Fehlings M.	33
67	Sonntag V.	Imagama S.	32
66	Ochi M.	Chikuda H.	31
*Top 10 authors by 2nd Order Connections*		*Top 10 authors by connected institutes*	
**Name of author**	**Second order connections**	**Name of author**	**Connected institutes**
Seichi A.	163	Seichi A.	38
Kato T.	148	Fehlings M.	33
Yamazaki M.	148	Riew K.	33
Kimura A.	148	Toyama Y.	33
Taguchi T.	143	Taguchi T.	31
Toyama Y.	142	Takeshita K.	30
Takeshita K.	141	Yamazaki M.	28
Chikuda H.	129	Chikuda H.	27
Kaito T.	129	Kato T.	27
Fujimori T.	129	Yonenobu K.	26
*Top 10 authors by betweenness*			
**Name of author**	**Betweenness**		
Riew K.	228.2296532		
Heller J.	93.6082689		
Oshima Y.	80.3588865		
Takeshita K.	71.41397157		
Yonenobu K.	62.18250124		
Seichi A.	60.53062928		
Taguchi T.	49.08174472		
Chikuda H.	48.14430465		
Imagama S.	48.09630067		
Miyauchi A.	45.9		

H-Index is explained in text. First order connections are the total number of an author’s co-authors. Second order connections are the total number of the co-authors’ co-authors. Connected institutes is the number of distinct research institutes in which the author had co-authors. Betweenness is a measure of how ‘central’ an author is to information flow within the network [22].

**Fig 3:**
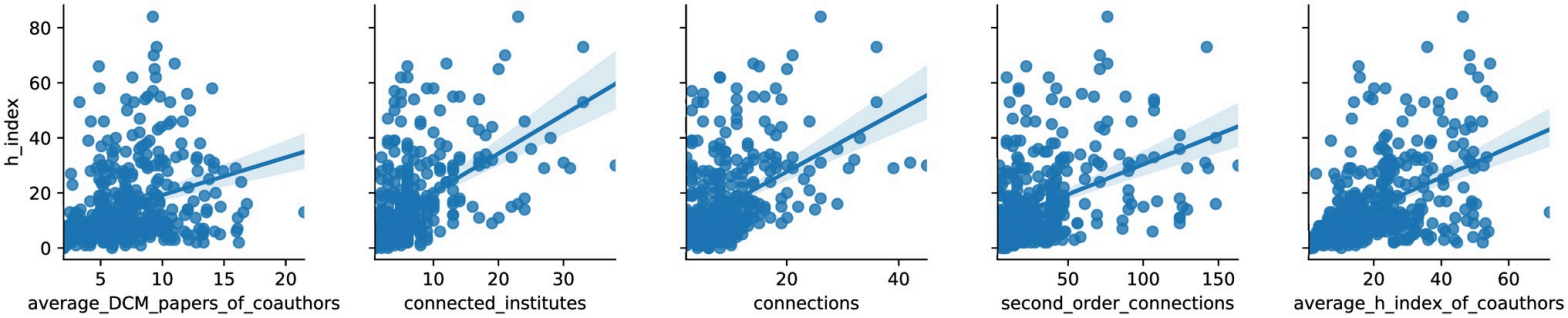
Distributions of various network metrics with H-Index. Average DCM papers of co-authors is the total number of papers an author’s co-authors have published. Connected institutes is the number of distinct research institutes in which the author had co-authors. Connections are the total number of an author’s co-authors. Second order connections are the total number of the co-authors’ co-authors.

Comparative distributions of author data with H-Index of interest, and data about the distributions of CN involvement metrics, are shown in Table 2 and Fig 3.

**Table 2:** Statistical data about authors in the co-authorship network.

	Mean	SD	Median	Max
Connected countries	*1*.*2*	0.6	1.0	5.0
Connected institutes	*6*.*2*	5.5	4.5	38.0
Average h index of co-authors	*20*.*3*	13.5	17.5	72.0
Average number of papers of co-authors	*6*.*9*	3.5	6.3	21.5
Total Network connections	*8*.*4*	6.2	7.0	45.0
Connections per paper	*2*.*1*	1.2	1.8	9.5
Second order connections	*29*.*2*	30.4	16.0	163.0
Second order connections per paper	*7*.*1*	7.3	4.5	62.0
Third order connections	*68*.*3*	65.9	3.0	9.0
Third order connections per paper	*17*.*0*	19.9	0.6	3.0
Betweenness	*3*.*3*	14.5	0.0	228.2
Closeness	*2*.*7*	0.9	3.5	3.5

Each row contains one of the “network involvement factors” used to predict author impact. “Betweenness” and “Closeness” are standard measures of node centrality in network theory [22, 23]. Connections refer to the number of authors a given author is connected to in the network.

### Machine learning

All numbers in this section are approximate, as the neural network was randomly initialised, and trained on a random 80% of authors. Re-running the code produces results within a 1% variance. All code used in this paper is available in the following GitHub repo: https://github.com/noahgrodzinski/DCM_network_analysis_repo.

#### Network learning

With the co-authorship network set up as described, and the authors selected, the neural network was trained at a learning rate of 0.0001. Higher and lower learning rates were found to be less effective.

#### Predictive accuracy & statistical significance

**Fig 4:**
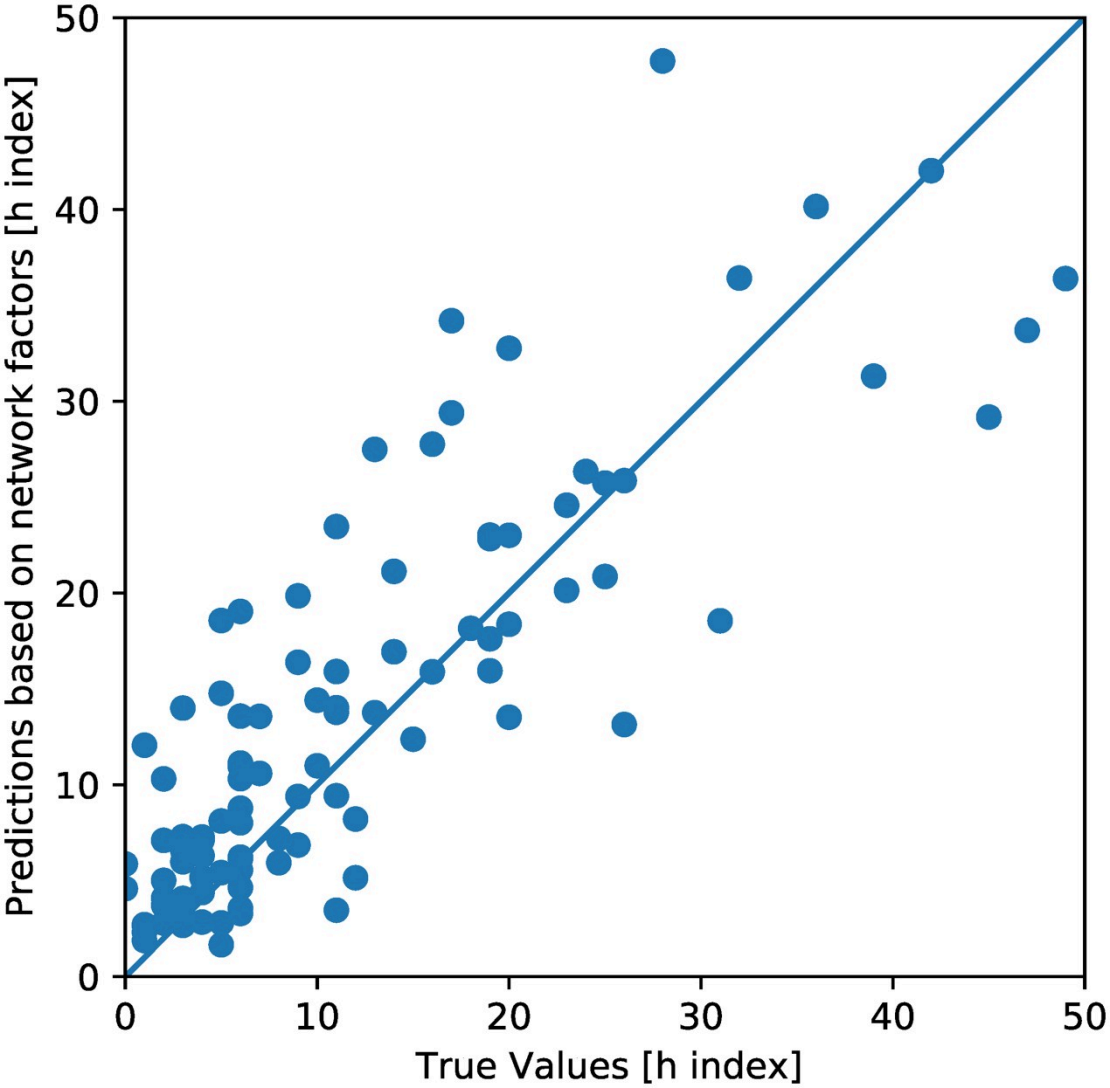
True and predicted values for H-Index. Predictions up to an H-Index of 50 are shown.

When retrospectively validated on the 20% of authors (109 authors) “set aside” (i.e., unseen by the network), the predictions made by the neural network had a Spearman’s correlation coefficient of 0.85 (Fig 4). There was a probability of p = 3.5866286 *10^−32^ of the correlation being due to chance.

**Fig 5:**
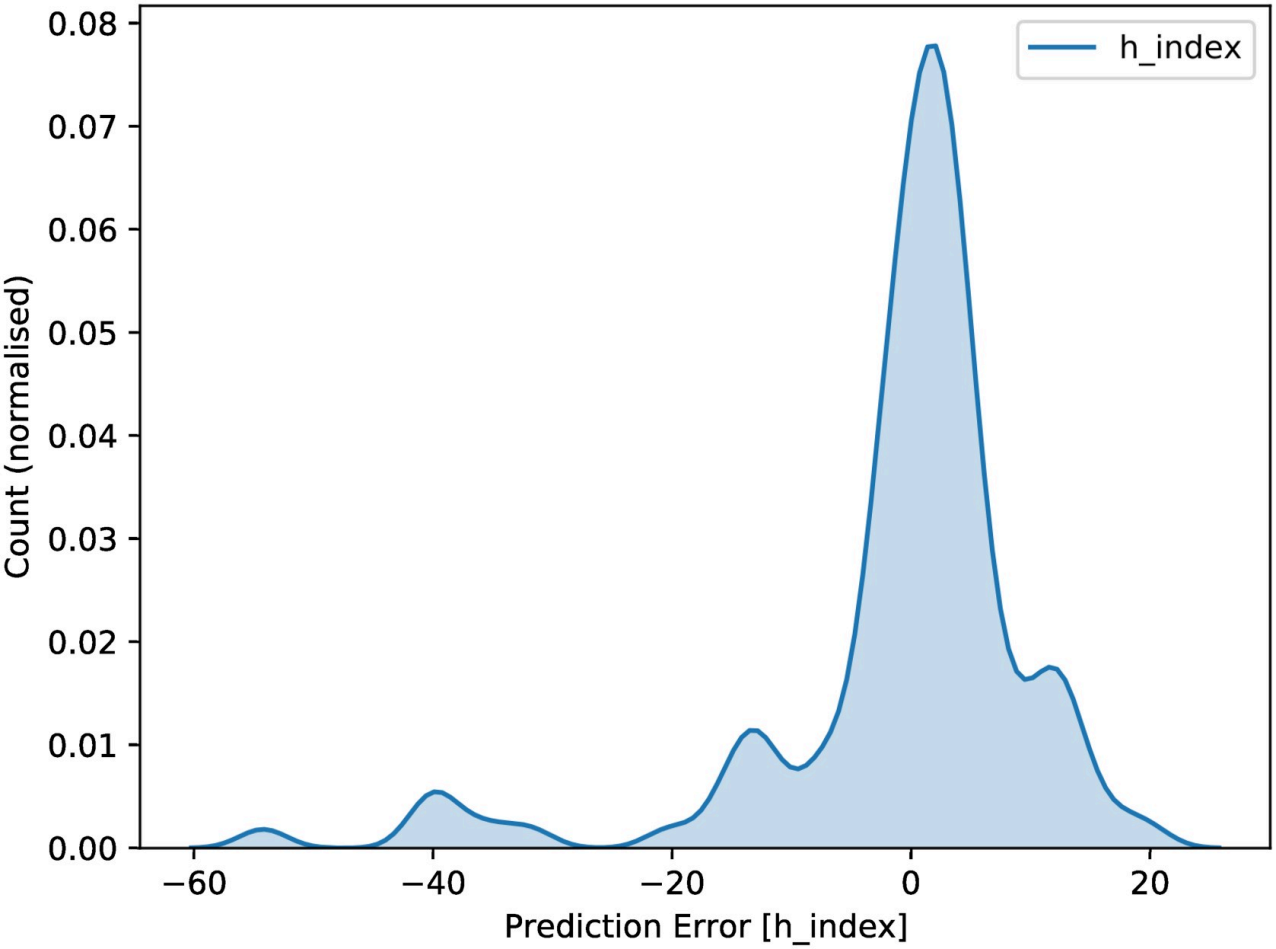
KDE plot of h-index prediction error. The prediction error is the difference between the true and predicted value. Thus, a negative number shows the network under-predicted, a positive value showed the network over-predicted.

Using the absolute values of error, the median prediction error was 3.33, the mean prediction error was 7.08 (i.e., the prediction for h index was an average of 7.08 units off), and the standard deviation for predictive error was 9.50. Using relative values of error, the median prediction error was 38.6% from the true value (Fig 5).

#### Network sensitivities

**Fig 6:**
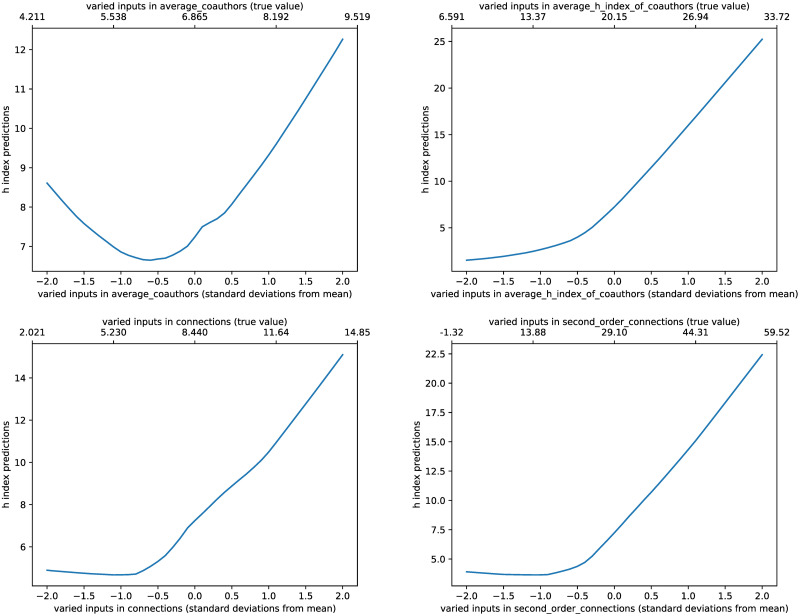
Variation in predicted H-Index with author connectedness statistics. The bottom axis shows the standard deviations from the mean; the top axis shows the actual number.

The network was optimised to recognise patterns which indicate high author impact (through the surrogate of H-Index). By varying inputs, we identified the patterns that the network recognises as leading to the highest impact research (Fig 6).

Firstly, the effect of number of co-authors on author impact prediction. Large group collaborations (around 9 people) tend to lead to high impact research, as do very small group collaborations (around 4 people). However, collaborations of around 5–7 people, the most common research group size, seems to have the lowest impact prediction.

Secondly, the effect of co-author impact on author impact prediction. Unsurprisingly, the network also predicts that authors who have collaborate with high-impact co-authors tend, themselves, to have higher H-Indexes.

Finally, the effect of co-authorship links on author impact prediction. Authors more well-connected to the rest of the co-authorship network were predicted to have higher H-Indexes. This increase in h-index prediction is exaggerated for second-order connections (reaching up to 22.5 at 2 standard deviations from the mean value, instead of 15). This suggests that the neural network uses second order connections as a stronger predictor of author impact than first order connections.

## Discussion

This is the first study to explore a researcher’s impact, within a neurological research environment, using artificial intelligence. The study reconfirms that DCM researchers are geographically clustered, with collaboration often between centres, but seldom across borders. Further whilst the accuracy of the neural network was less than 100%, with a median error of 3.3 units of H-Index, indicating other factors are relevant, the strong predictive power of the network metrics in retrospective validation demonstrates the significance of collaboration in determining impact.

Specifically, on a statistical level, the following insights are identified. Firstly, authors who collaborate with high-impact co-authors tend themselves, to have a higher predicted H-Index. Secondly, authors who collaborate in small groups <4 authors, and authors who work in very large groups >10, tended to have the highest predictions for H-Index. Further, second order connections have a stronger positive influence on H-Index prediction than first order connections, indicating that collaborations with better connected co-authors are more valuable than the number of collaborations.

In practical context, these observations indicate both the value of hierarchical research structures, which commonly foster most research training and mentorship today [24], but also reinforce the value for research visibility of identifying agents of change with a greater connection to the whole research network.

In this regard, the use of network statistics and artificial intelligence are likely to add value. The only data provided to our machine learning algorithm on each author was their network metrics, the set of values associated with a given author’s connections within the co-authorship network. This means the impact predictions of each author, as generated by the machine learning algorithm, are based only on the authors connectivity in the co-authorship network.

This has two advantages. Firstly, it means that the machine learning algorithm could just as easily be trained on any other author impact metric (not just H-Index) relevant to a given field and makes the network a highly versatile tool. Secondly, the algorithm is likely to give a more valid representation of an author’s impact within a specific research field, given that its predictions are a function of the author’s positioning within the research community, which has an intuitive role in research impact–i.e., its predictions are justifiable on non-positivist grounds. This is not accounted for using traditional bibliometrics (e.g., H Index) which may be influenced by other research interests or self / localised citation [10].

### Would greater collaboration benefit DCM?

One of the principles that led to the AO Spine RECODE-DCM (aospine.org/recode) initiative [6] was a lack of synergy between the global DCM research community. This was demonstrated through the overlap and duplication of research [15], the inconsistent use of terminology [25] and selection or reporting of data elements including outcomes [13, 14]. A suggested basis for this was a relative lack of diversity, including an underrepresentation of non-surgeons, fundamental stakeholders in DCM care [15, 26], and patient perspective [27, 28]. However, the limited interconnectivity of the research network demonstrated here is likely a further and key contributor. It is of note, the average number of authors per paper in DCM research was 7.9, well under the life-sciences 2016 average of 13.7 [29].

For a field in urgent need of knowledge that can improve outcomes, and relative to conditions such as traumatic spinal cord injury or multiple sclerosis, with a significant lack of research activity or investment [30], the question arises—could increasing collaboration therefore be a key intervention for supporting DCM research?

Within DCM, the relative impact [8] of a few, highly connected research networks (e.g., those associated with Fehlings M., Riew K., and Seichi A.) would support this hypothesis, having either led, or significantly contributed to many of the landmark initiatives so far, such as the AO Spine North America [31], AO Spine International [32] and CSM Surgery [33] trials, alongside clinical practice guidelines [34]. These research networks could serve as a model for other groups to follow.

The value of scientific collaboration is multi-faceted, and not simply of value to visibility as mentioned so far. Collaboration can diversify skills and perspective, as well as improve and increase access to data [35, 36]. Broader analysis of life sciences supports the overall notion of increased collaboration [37–39], but there can be challenges [40] and a number of frameworks have been proposed to support this [35].

However, this should not dismiss or undermine the potential value of more focused science. As aforementioned, an increased number of co-authors was associated with a higher impact, this relationship was not linear, with small groups <4 authors having amongst the highest predictions for H-Index. This observation may be an artefact of the data, but is in line with workforce productivity studies, which show optimum efficiency for small group sizes [41], offset by increased collaboration in large groups [42]. Whilst this comparison may not be applicable to the present study, it contextualises the complex role of collaboration in research impact.

### Limitations

It should be acknowledged that our specific approach in this study has some limitations.

Firstly, validation was performed using a partition within the dataset, rather than a prospective set of collected data. The dataset was formed of primary clinical research, and did not contain pre-clinical only studies. Secondly, the neural network was trained on H-Index. This is not an ideal impact metric (for reasons already outlined), but was included as a widely used, quantitative surrogate of research impact. The versatility of our neural network means it could be trained on any other metric in future. However, there may be no quantitative metric that takes into account all factors that can influence impact, including publication journal, and personal relationships between authors. Thirdly, our analysis was performed using *current* H-Index. This was favoured pragmatically, to give immediate insight into potential agents of change, however, will overlook potential network changes, including early-career researchers. Further iterations of this methodology could consider using time-stamped metrics, with the advantage of incorporating metric change into the model.

Finally, the approach employed here focused on authors, with a view to identifying agents of change. This approach shows authors well-positioned within the present research network, but cannot accurately characterise individual authors’ real-life impact and or influence. Furthermore, whilst this is an average representation, this may not be directly supportive of visibility for a specific article. A network of papers, where a link represents a common author may produce insight on this and represents a follow-on line of enquiry.

## Conclusions

Within the field of DCM, research collaboration is largely clustered geographically, with limited connectivity across borders. Analysis of the co-authorship network allows identification of key opinion leaders well-positioned to aid knowledge translation. A machine learning algorithm was able to identify collaborative factors which influence research impact, which may be helpful in fostering optimised future collaborative networks.

## Supporting information

S1 Fig Interactive visualisation of co-authorship network for DCM research. Colours show country. Author node size shows number of papers published. Authors shown as lone nodes do not have any collaborations with the other “prolific” selected authors. Zooming in shows increasing levels of detail.
(HTML)
